# Return to Sport in Athletes with Midportion Achilles Tendinopathy: A Qualitative Systematic Review Regarding Definitions and Criteria

**DOI:** 10.1007/s40279-017-0833-9

**Published:** 2017-12-16

**Authors:** Bas Habets, Anke G. van den Broek, Bionka M. A. Huisstede, Frank J. G. Backx, Robert E. H. van Cingel

**Affiliations:** 1 Papendal Sports Medical Centre, Papendallaan 7, 6816 VD Arnhem, The Netherlands; 2 Department of Rehabilitation, Physical Therapy Science and Sports, Rudolf Magnus Institute of Neurosciences, University Medical Center Utrecht, Utrecht, The Netherlands; 3 Radboud University Medical Centre, Research Institute for Health Sciences, IQ Healthcare, Nijmegen, The Netherlands

## Abstract

**Background:**

Midportion Achilles tendinopathy (AT) can cause long-term absence from sports participation, and shows high recurrence rates. It is important that the decision to return to sport (RTS) is made carefully, based on sharply delimited criteria. Lack of a well-defined definition and criteria hampers the decision to RTS among athletes with AT, and impedes comparison of RTS rates between different studies.

**Objective:**

The aim of this study was to systematically review the literature for definitions of, and criteria for, RTS in AT research.

**Study Design:**

Qualitative systematic review.

**Methods:**

The PubMed, EMBASE, Cochrane, CINAHL, PEDro, and Scopus electronic databases were searched for articles that reported on the effect of a physiotherapeutic intervention for midportion AT. Article selection was independently performed by two researchers. Qualitative content analysis was used to analyze the included studies and extract definitions of, and criteria for, RTS.

**Results:**

Thirty-five studies were included in the content analysis, showing large variety in both the definitions and criteria. Thirty-two studies reported a definition of RTS, but only 19 studies described the criteria for RTS. The content analysis revealed that ‘reaching pre-injury activity/sports level, with the ability to perform training and matches without limitations’, ‘absence of pain’, and ‘recovery’ were the main content categories used to define RTS. Regarding the criteria for RTS, eight different content categories were defined: (1) ‘level of pain’; (2) ‘level of functional recovery’; (3) ‘recovery of muscle strength’; (4) ‘recovery of range of motion’; (5) ‘level of endurance of the involved limb’; (6) ‘medical advice’; (7) ‘psychosocial factors’; and (8) ‘anatomical/physiological properties of the musculotendinous complex’. Many criteria were not clearly operationalized and lacked specific information.

**Conclusions:**

This systematic review shows that RTS may be defined according to the pre-injury level of sports (including both training and matches), but also with terms related to the absence of pain and recovery. Multiple criteria for RTS were found, which were all related to level of pain, level of functional recovery, muscular strength, range of motion, endurance, medical advice, psychosocial factors, or anatomical/physiological properties of the Achilles tendon. For most of the criteria we identified, no clear operationalization was given, which limits their validity and practical usability. Further research on how RTS after midportion AT should be defined, and which criteria should be used, is warranted.

**PROSPERO Registration Number:**

CRD42017062518.

**Electronic supplementary material:**

The online version of this article (10.1007/s40279-017-0833-9) contains supplementary material, which is available to authorized users.

## Key Points

**Table:** 

There appears to be large variation in how return to sport (RTS) after midportion Achilles tendinopathy (AT) is defined within the current literature.
Numerous criteria for RTS are proposed, but the majority of these criteria lack clear operationalization and cut-off values.
There is a strong need for clinicians and researchers to reach consensus on a clear definition and criteria for RTS after midportion AT.

## Introduction

Midportion Achilles tendinopathy (AT) can cause a prolonged absence from sports participation, and may even be career-ending in up to 5% of athletes with AT [1]. Recurrence rates as high as 27% have been reported, particularly in those with short recovery periods (0–10 days) [2], which might be related to the fact that, although symptoms have fully subsided, deficits in musculotendinous function may still persist in 25% of patients, putting the athlete at risk for re-injury [3]. Therefore, it is important that a decision on return to sport (RTS) is carefully made, based on multiple factors and involving all relevant stakeholders [4].

In a recent systematic review on eccentric training for midportion AT, performed by our research group [5], we found that only one-third of the included studies used RTS as an outcome, with a RTS rate ranging between 10 and 86% after 12 weeks [6, 7]. These studies used different definitions (e.g. ‘return to previous activity level’ or ‘return to full activity’), which makes comparison of their RTS rates difficult. In many other AT studies, RTS is either not the main outcome of the study or is not evaluated at all. This results in a lack of clear definition of RTS and an absence of well-defined criteria for RTS.

In 2016, a consensus statement on RTS after sports injuries was developed [4] which stated that “the definition of each RTS process should, at a minimum, be according to the sport […] and the level of participation […] that the athlete aims to return to” [4]. Silbernagel and Crossley [8] recently proposed a program aimed at RTS for athletes with midportion AT. While this program provides a useful rationale and progression to RTS, unfortunately the authors did not explicitly report a single clear definition of RTS, or the exact criteria that should be met.

The lack of a clear definition and well-defined criteria can hamper the decision making for RTS among athletes with AT. Moreover, it impedes comparison of RTS rates between different intervention studies. Therefore, the aim of this review was to systematically analyze the current literature for definitions of RTS in AT research, and investigate which criteria for RTS are being used.

## Methods

### Study Design

This systematic review was developed in accordance with the Preferred Reporting Items for Systematic Reviews and Meta-Analyses (PRISMA) guidelines, and was prospectively registered in the PROSPERO database for systematic reviews (registration number CRD42017062518).

The purpose of the study was twofold: (1) to synthesize definitions of RTS, where RTS was seen as a successful endpoint after midportion AT; and (2) to search for criteria used in scientific literature for decision making to initiate RTS.

### Search Strategy

A systematic search of the literature from 1998 to July 2017 was conducted in the PubMed, EMBASE, Cochrane, CINAHL, PEDro, and Scopus electronic databases. The search was limited from 1998 onwards based on the paper from Maffulli et al. [9]. According to this paper, the terminology changed from ‘tendinitis’, considered as a frank inflammation of the tendon, to ‘tendinopathy’, which is a combination of frequent longstanding pain, swelling and impaired performance [9]. This paradigm shift has led to changes in the management of tendinopathic injuries (i.e. targeted more at reducing symptoms and increasing load capacity rather than minimizing inflammation using non-steroid medication and/or injections), and this can have consequences for the factors associated with the RTS decision.

The search strategy contained various synonyms for ‘Achilles tendinopathy’. For ‘return to sport’, we partially adopted a search strategy used in a similar research on return to play after hamstring injuries [10], and modified this to fit our study purpose. The final search strategy can be found in electronic supplementary Appendix S1.

### Eligibility Criteria

All retrieved articles were independently screened for eligibility by two authors (BHa, AvdB). All studies investigating the effect of any physiotherapeutic intervention in an adult (≥ 18 years) athletic population (i.e. individuals who participate in organized or non-organized sports) with midportion AT were eligible for inclusion if they (1) described a definition of, and/or criteria for, RTS, and (2) were written in English, Dutch or German. There were no restrictions on type of study design. Articles that adopted definitions from other studies were excluded, but the studies from which the original definition was adopted were screened for eligibility, and included when they met our eligibility criteria. Potential articles were further excluded if they (1) were not available in full-text, despite serious efforts to contact the corresponding author; (2) described interventions for insertional AT and/or Achilles tendon rupture; (3) investigated surgical or other invasive interventions; or (4) were animal studies.

A consensus meeting between the two authors was held to discuss discrepancies in article screening and selection. If no consensus could be reached between the two authors, a third author (BHu) was asked to make a final decision. Cohen’s kappa was calculated to indicate agreement between the two authors. A Cohen’s kappa > 0.61 was considered as substantial agreement.

### Data Extraction

Two authors (BHa, AvdB) performed the data extraction from the included studies, using a standardized extraction form. The following relevant data were extracted: (1) first author; (2) year of publication; (3) study design; (4) study population, type and level of sport; (5) definition of the diagnosis of AT; (6) definition of RTS; (7) criteria described for initiation of RTS; and (8) recurrence rate and residual symptoms.

### Data Analysis

We searched for definitions of, as well as criteria for, RTS using a content analysis approach [11–13]. This is a qualitative method, aimed at classifying the written material into identified categories in three steps [14]. The first step of content analysis is open coding [15]. Two researchers (BHa, AvdB) independently read through the included studies several times and started to identify provisional labels by making notes in the text indicating text fragments/aspects related to definitions of, or criteria for, RTS. A consensus meeting was conducted to compare the results of this step and discuss potential discrepancies.

The second step is axial coding, which aims to explore the relationships/associations among the provisional labels identified by open coding [15]. Both authors (BHa, AvdB) independently performed the axial coding process, and a consensus meeting was held afterwards to discuss potential discrepancies.

The third step of content analysis is selective coding [15]. During this step, the researchers aimed to develop overarching content categories that serve as umbrella terms for the labels identified during the axial coding phase. In the current review, the selective coding phase resulted in an overview of relevant terms that are used to define RTS after midportion AT, and the criteria that are used for the RTS decision.

## Results

### Search Results

**Fig. 1:**
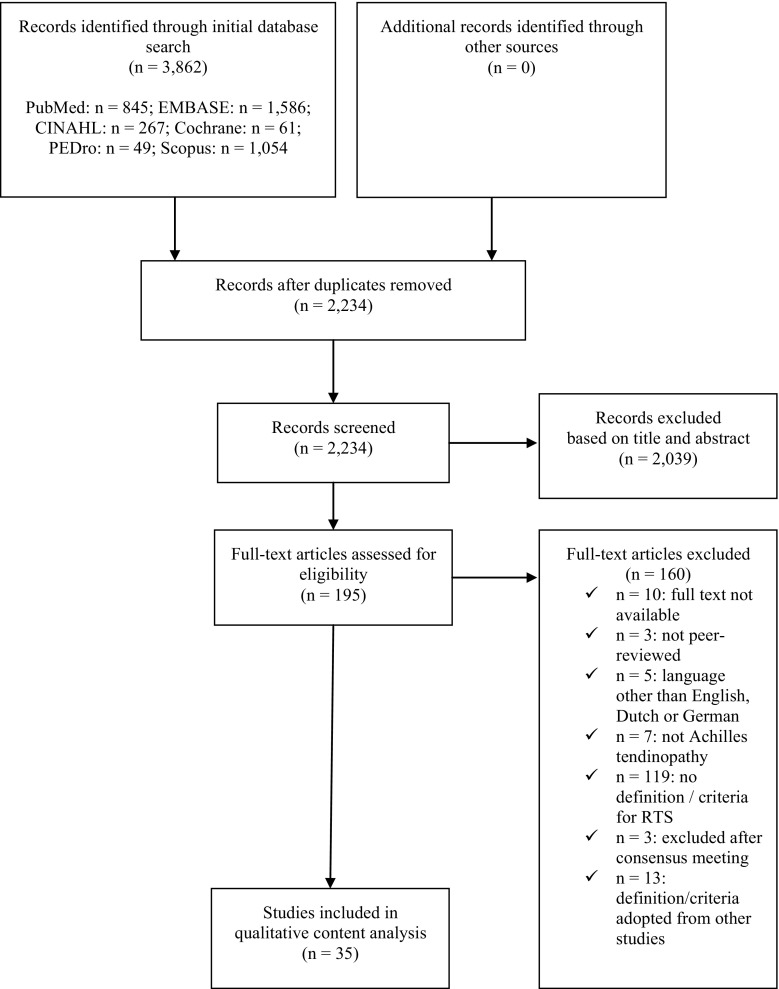
Study search strategy. *RTS* return to sport

The initial search yielded 3862 hits (Fig. 1). After removal of duplicates, 2234 potential articles remained for inclusion. Screening of the titles and abstracts resulted in exclusion of another 2039 articles, leaving 195 articles for full-text assessment. Of these, 10 (5%) could not be obtained in full-text, despite repeated attempts to contact the corresponding author by e-mail or through ResearchGate, and despite attempts to purchase a copy. One hundred and thirty-four studies were excluded after full-text assessment. No consensus was reached on the eligibility of five articles. After consulting our third author (BHu), the studies by Cook et al. [16] and Herrington and McCulloch [17] were included, while three other studies were excluded as they did not provide a definition of, or criteria for, RTS.

Forty-eight articles met our inclusion criteria, but another 13 were excluded as they used a definition that was adopted from other studies. The studies containing the original definition were already included, therefore this resulted in a total of 35 articles that were included in the qualitative content analysis. These 35 studies included 10 randomized controlled trials, two non-randomized controlled trials, four pre-post studies, two retrospective cohort studies, one case series, two case studies, eight narrative reviews, four clinical commentaries, one masterclass report, and one guideline report.

At this stage, Cohen’s kappa was 0.69, indicating substantial agreement [18].

### Content Analysis

#### Definition

**Fig. 2:**
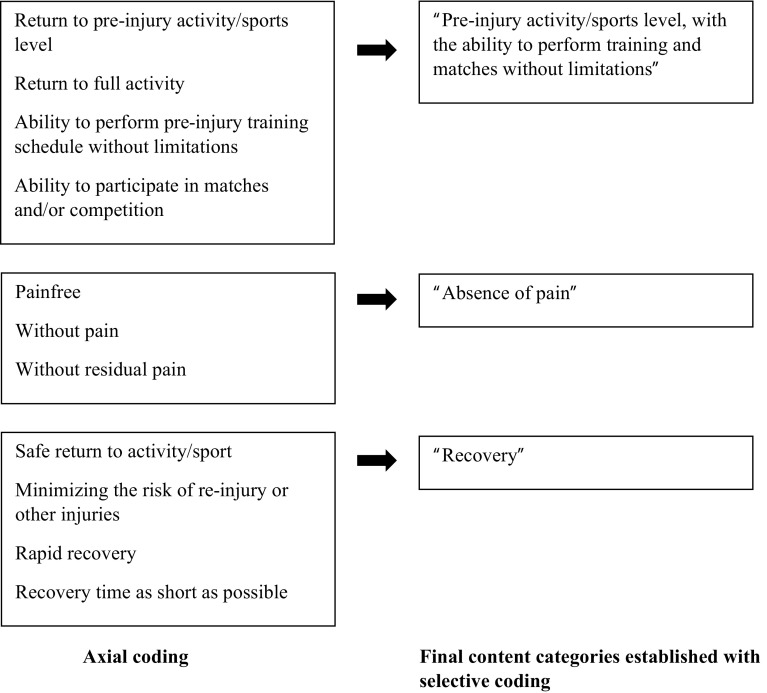
Axial coding and selective coding of the content analysis for the definition of return to sport after midportion Achilles tendinopathy

Of the 35 included studies, 32 (91%) provided one or multiple definitions of RTS for athletes with midportion AT. These definitions were extracted during the open coding phase of the content analysis (Table 1). During the axial coding phase, several categories were formed, which subsequently were grouped into three distinct content categories in the selective coding phase. These content categories were ‘pre-injury activity/sports level, with the ability to perform training and matches without limitations’, ‘absence of pain’ and ‘recovery’ (Fig. 2).

**Table 1:** Definitions of, and criteria for, return to sport, as described in the included studies (similar to open coding of the content analysis)

Study (year of publication)	Design	Population; *n*, sex; average age ± SD level and type of sport	Diagnosis	Definition of RTS	Criteria for RTS	RR residual symptoms
Alfredson et al. [43] (1998)	CCT	15 Athletes; 12 F, 3 M; 44.3 ± 7.0 years Recreational running 15 Athletes; 4 F, 11 M; 39.6 ± 7.9 years Recreational running and soccer	Achilles tendinosis: pain located in the Achilles tendon (2–6 cm above insertion on the calcaneus) for at least 3 months	Back at their pre-injury levels with full running activity Resumption of previous running activity	Running activity was allowed if it could be performed with only mild discomfort and no pain	RR not reported VAS mean 4.8/10 after returning to pre-injury activity level
Alfredson and Cook [20] (2007)	Narrative review	NA	Achilles tendinopathy: Achilles mid-tendon pain, focal or generalized swelling	Back to previous tendon-loading activity level Back to previous activity level	Not reported	Both not reported
Ammendolia et al. [51] (2016)	RCT	19 Athletes; sex not reported; 28.3 ± 4.9 years 16 athletes; sex not reported; 28.8 ± 4.4 years Elite volleyball	Overuse Achilles tendinitis	Resumption of training in the gym; Return to play volleyball	Not reported	RR not reported VAS mean 3.8–4.9 when return to training in the gym VAS mean 0.6–2.4/10 when RTS
Barry [33] (2010)	Case study	1 M; 40 years Recreational running	Achilles tendinopathy	Returns to his training schedule without limitations	Not reported	Both not reported
Beyer et al. [22] (2015)	RCT	25 Recreational athletes; 7 F, 18 M; 48 ± 2 years 22 recreational athletes; 8 F, 14 M; 48 ± 2 years Recreational level, type not reported	Chronic unilateral midportion Achilles tendinopathy, based on defined clinical findings (VISA-A score and VAS scale), physical examination, and pain duration of at least 3 months; and US findings needed to be present, i.e. local A-P thickening of the midtendon level, with a hypoechoic area and a color Doppler signal within the hypoechoic area	Resumed their previous activity levels	Sporting activities should be performed with a discomfort not exceeding 30 mm on the VAS	Both not reported
Biedert et al. [42] (2006)	Clinical commentary	NA	Not reported	Return to physical fitness/former sport activities. Physical fitness can be divided into general and sports-specific physical fitness The authors further describe a stepwise progression from sports-specific training to match-specific training to match training	The return to former sports activities depends on different factors such as structural healing, functional re-integration, physical examination, and specific investigations and tests, as well as individual goals and mental aspects	Both not reported
Chazan [30] (1998)	Narrative review	NA	Achilles tendinitis, Achilles tendinosis	Return/resumption to full activity	Not reported	Both not reported
Chessin [41] (2012)	Narrative review	NA	Achilles tendinitis: an inflammation of the tendon Achilles tendinosis: a chronic, non-inflammatory condition that is consistent with degenerated tissue and disorganized tendon structure	Not reported	Capable of maintaining full dynamic load and controlling directional and speed changes with confidence. This requires progressive training for a balance of strength and flexibility, as well as building endurance and proprioceptive control	Both not reported
Chinn and Hertel [37] (2010)	Narrative review	NA	Achilles tendonitis: an inflammatory condition that involves the Achilles tendon and/or its tendon sheath. Typically, the athlete will suffer from gradual pain and stiffness in the Achilles tendon region, 2–6 cm proximal to the calcaneal insertion	Full participation at full functioning Full competition Graduated return to physical activity Safe return to sport while minimizing the risk of recurrent injuries	Athletes should be allowed to compete when full range of motion and strength has returned. The athlete should have regained endurance in the involved limb and be capable of completing full practice without pain	Both not reported
Cook et al. [16] (2002)	Masterclass report	NA	Not reported	Return to training and competition	Inadequate amounts of load, speed and endurance may result in incomplete rehabilitation and insufficient musculotendinous function to return to sport	Both not reported
De Vos et al. [29] (2007)	RCT	32 Athletic patients; 12 F, 20 M; 44.1 ± 7 years 31 athletic patients; 14 F, 17 M; 45.1 ± 8.9 years Recreational level, type not reported	Achilles tendinopathy: a tendon that was tender on palpation and painful during or after sport. The tendon thickening was located approximately 2–7 cm proximal to the distal insertion. Diagnosis was based on clinical examination	Return to their original level of sports	After 4 weeks, gradual return to sports activities was encouraged if the pain allowed it	Both not reported
Dijkstra and Van Enst [52] (2003)	Retrospective cohort study	9 Patients; 4 F, 5 M; 43.2 years (range 26–65) Level not reported, athletics (*n* = 6)	Achilles tendinosis; diagnosis based on history and clinical examination	Fully functional at the original sports level	Not reported	Both not reported
Fahlström et al. [19] (2003)	Pre-post study	78 patients; 25 F, 53 M; 46.1 ± 9.5 years Recreational level, running, walking and other sports	Chronic painful Achilles tendinosis at the midportion of the tendon (2–6 cm from the tendon insertion), with a duration of at least 3 months. Diagnosis based on clinical examination (painful nodular thickening of the Achilles tendon located at the level 2–6 cm from the tendon insertion) and US (local thickening of the tendon, irregular structure with hypoechoic areas and irregular fiber orientation)	To return to previous (before injury) activity level Come back to previous (before injury) activity level To be able to participate in his/her desired sports/recreational activities Be fully active in their sport	During the 12-week training regimen, jogging/walking activity was allowed if it could be performed with only mild discomfort and no pain Patients were instructed to start jogging or walking at a slow pace, on flat ground, and for a short distance. Thereafter, their activity could be gradually increased if there was no severe pain in the tendon (similar to Mafi et al. 2001 [7])	RR not reported VAS mean 10.2/100 after returning to previous activity level
Giombini et al. [26] (2002)	RCT	44 Athletes; 11 F, 33 M; 26.0 ± 4.6 years Competitive level, type not reported	Achilles tendinopathy: pain and tenderness on palpation at the midportion of the tendon or at the distal insertion, associated with tendon swelling (diffuse or localized)	Full return to their pre-injury sport level A full return to sport Return to specific sport activity	Not reported	RR not reported ~ 25% of athletes reported occasional discomfort after RTS
Herrington and McCulloch [17] (2007)	RCT	13 Patients; sex not reported; 37.0 ± 9.3 years 12 patients; sex not reported; 36.6 ± 7.1 years Achilles loading sports, level and type not specifically reported	Non-insertional Achilles tendinopathy; local Achilles pain, stiffness or functional impairment on activity	Full return to the desired level of activity Full return to activity Returned to their previous activity levels	Not reported	Both not reported
Kountouris and Cook [23] (2007)	Narrative review	NA	Achilles tendinopathy	Return to pre-injury levels of activity Return to competition	To achieve return to pre-injury activity levels, rehabilitation program must incorporate some general principles of exercise program design, such as strength, endurance, power, and a gradual return to sports-specific function	Both not reported
Lakshmanan and O’Doherty [28] (2004)	Pre-post study	15 Patients (16 tendons); 3 F, 12 M; 48.5 years (range 35–77) Active sports, level and type not specifically reported	Chronic non-insertional Achilles tendinopathy, for more than 6 months; diagnosis confirmed by US	Return back to their normal activities Return to full training activities with no limitation Returning back to the original level of physical activity in active sports persons Return back to their sports activities	Not reported	Both not reported
Langberg et al. [53] (2007)	CCT	6 Elite soccer player patients; 6 M; 26 ± 1 year (the non-injured tendon served as a control) Elite soccer	Unilateral Achilles tendinosis: pain 30–60 mm above the Achilles tendon insertion on the calcaneus	Return to the previous level of physical activity Back playing soccer	Subjects were allowed to continue soccer training if the pain had not increased	RR not reported VAS mean 13/100 after resuming soccer
Mafi et al. [7] (2001)	RCT	22 Patients; 10 F, 12 M; 48.1 ± 9.5 years 22 patients; 10 F, 12 M; 48.4 ± 8.3 years Recreational level, jogging and walking	Painful chronic Achilles tendinosis located at the 2–6 cm level in the tendon. Diagnosis based on clinical examination and US	Resumed their previous activity level (before injury)	During the 12-week training regimen, jogging/walking activity was allowed if it could be performed with only mild discomfort and no pain Patients were instructed to start jogging or walking at a slow pace, on flat ground, and for a short distance. Thereafter, their activity could be gradually increased if there was no severe pain in the tendon	RR not reported VAS mean 9–12/100 after resuming previous activity level
McShane et al. [34] (2007)	Narrative review	NA	Non-insertional Achilles tendinopathy	Pain-free return to activity Back to their pre-injury level training regimen Returned to pre-injury training levels	Not reported	Both not reported
Nicola and El Shami [35] (2012)	Clinical commentary	NA	Midportion Achilles tendinopathy	Return to running without pain	Daily activities should be pain-free before returning to training For soft tissue injuries, there should be minimal residual tenderness In general, a period of 1–2 weeks of pain-free daily activities should be present before any consideration of return to running No running until patient is able to walk comfortably at 4.0 mph for 10 miles per week	Both not reported
Paavola et al. [24] (2000)	Pre-post study	83 Patients; 22 F, 61 M; 32 ± 11 years Competitive and recreational level, running and orienteering	A diagnosis of unilateral, non-chronic Achilles tendinopathy based on clinical examination (defined as exertional pain and palpable tenderness in the Achilles tendon of < 6 months’ duration)	Returned to their pre-injury level of physical activity Fully recovered their physical activity level	Not reported	Both not reported
Paavola et al. [36] (2002)	Review	NA	Combination of Achilles tendon pain, swelling, and impaired performance	To return the patient to the desired level of physical activity without residual pain. In athletes, an additional demand is that the recovery time should be as short as possible Able to return to full levels of physical activity	Not reported	Both not reported
Petersen et al. [25] (2007)	RCT	37 Patients; 14 F, 23 M; 42.5 ± 11.1 years 35 patients; 15 F, 20 M; 42.6 ± 10.7 years 28 patients; 11 F, 17 M; 43 ± 12 years Recreational level, running, walking and other sports	Gradually evolving painful condition in the Achilles tendon located at the midportion, for at least 3 months; diagnosis based on clinical examination and US	Return to pre-injury sports level Full recovery to previous activity level	Jogging, walking and cycling were allowed if they could be performed with only mild discomfort or pain	Both not reported
Rompe et al. [31] (2007)	RCT	25 Patients; 16 F, 9 M; 48.1 ± 9.9 years 25 patients; 14 F, 11 M; 51.2 ± 10.3 years 25 patients; 16 F, 9 M; 46.4 ± 11.4 years Athletic patients, level and type not specifically reported	Pain over the main body of the Achilles tendon 2–6 cm proximal to its insertion, swelling and impaired function; clinical examination and US	Return to their normal levels of activity Return to full activity is possible	During the 12-week training regimen, jogging/walking activity was allowed if it could be performed with only mild discomfort and no pain Patients were instructed to start jogging or walking at a slow pace, on flat ground, and for a short distance. Thereafter, their activity could be gradually increased if there was no severe pain in the tendon (similar to Mafi et al. 2001 [7])	Both not reported
Rompe et al. [21] (2009)	RCT	34 Patients; 20 F, 14 M; 46.2 ± 10.2 years 34 patients; 18 F, 16 M; 53.1 ± 9.6 years Athletic patients, level and type not specifically reported	Pain over the main body of the Achilles tendon 2–6 cm proximal to its insertion, swelling and impaired function; clinical examination and US	Return to full activity Return to their previous sports/recreational activity level	During the 12-week training regimen, jogging/walking activity was allowed if it could be performed with only mild discomfort and no pain Patients were instructed to start jogging or walking at a slow pace, on flat ground, and for a short distance. Thereafter, their activity could be gradually increased if there was no severe pain in the tendon (similar to Mafi et al. 2001 7)	Both not reported
Roos et al. [6] (2004)	RCT	44 Patients; 23 F, 21 M; 46 years (range 26–60) Active in sport, level and type not specifically reported	Pain and swelling 2–6 cm proximal of the Achilles tendon insertion	Returned to their pre-injury activity level	Not reported	RR not reported 27–50% of patients reported moderate to extreme difficulties after returning to pre-injury activity level
Ross et al. [54] (2018)	Case report	1 M; 23 years Semi-professional volleyball	Site of maximal tenderness 4 cm proximal to the Achilles insertion; clinical examination	Return to peak performance Return to volleyball Return to professional sport performance	Not reported	Both not reported
Silbernagel et al [39]. (2011)	Case series (follow-up of an RCT)	34 Athletes; 16 F, 18 M; 51.0 ± 8.2 years Recreational level, type not specifically reported	Clinical diagnosis of a combination of Achilles tendon pain, swelling and impaired performance	Not reported	LSI below the level of 90% often used as a guideline for return to sports	5/34 Patients reported recurrence of symptoms after 5 year follow-up Symptoms not reported
Silbernagel and Crossley [8] (2015)	Clinical commentary	NA	Overuse injury, characterized by a combination of pain, swelling (diffuse or localized) and impaired performance; midportion Achilles tendinopathy is located 2–6 cm proximal to the insertion of the tendon on the calcaneus; based on history and physical examination	Return to sport with a low risk of re-injury or risk for other injuries Return to sport and previous activity level Back to sport participation Full return to sports Full sports participation Return to full sports activity	Resumption of activities such as running and jumping is generally recommended when the symptoms have subsided There are various factors that need to be considered when planning a return to sport after Achilles tendinopathy. The most obvious factor is the level of pain with physical activity. Other important factors that need to be included in the decision-making process are the healing and recovery of the tendon tissue, the recovery of strength, range of motion, and function, as well as the demands of the specific sport Return-to-sport activity may be started prior to complete absence of symptoms Addressing calf muscle weakness and/or muscle imbalance, and altered joint mobility of the foot and ankle complex, with the aim of regaining full capacity, is important for athletes prior to full sports participation Return to full sports activity should involve gradual loading progression Knowledge of the rate and magnitude of Achilles tendon loads Before an athlete is allowed to return to any running or jumping activity, he or she should have minimal (1–2/10 on the NPRS) to no pain with all activities of daily living The return-to-sport program is initiated when the athlete meets the requirement of performing activities of daily living with pain no higher than 2/10	Both not reported
Sorosky et al. [44] (2004)	Clinical commentary	NA	The combination of pain, swelling and impaired performance	Not reported	During the functional phase, jogging should be introduced gradually, and increased only when there is no pain during or after exercise	Both not reported
Van Linschoten et al. [27] (2007)	Guideline report	NA	Not reported	Return to the original level of sports	Not reported	Both not reported
Verrall et al. [32] (2011)	Retrospective cohort study	190 Patients; 82 F, 108 M; 39 years Running and walking (*n* = 108), level not specifically reported	Tenderness on palpation and visible swelling of the mid-substance of the Achilles tendon; based on clinical assessment	Return to their preferred activity/sport Return to full activity Resumed unrestricted activity Resuming full activity but with some ongoing symptoms Return to their physical activity	Not reported	RR not reported 21% of patients had ongoing symptoms after return to full activity
Werd [38] (2007)	Narrative review	NA	Not reported	Promptly returning to activity and avoiding repeated injury Safe and rapid return to activity Returning an injured athlete to sports as quickly and safely as possible	Return-to-play decisions should be based on an absence of pain, strength and range of motion equal to those of the contralateral limb, a gradual stepwise training protocol, and the ability of the athlete to perform the necessary skills of the sport without restriction	Both not reported
Wetke et al. [40]. (2014)	Pre-post study	93 Patients; 43 F, 52 years (range 18–73) 50 M, 46 years (range 21–73) Active in sports, level and type not specifically reported	Local tenderness at palpation of tendon, tenosynovium or tendon insertion impairing the daily activities of the patient; clinical examination and US	Back to their former sports activity	All jumping and running exercises were paused until the patient could do 20 one-legged heel lifts on the stairs, in three series, without increased pain, and then walking/running activities were slowly resumed	Both not reported

*RTS* return to sport, *RR* recurrence rate, *SD* standard deviation, *CCT* clinically controlled trial, *F* female, *M* male, *NA* not applicable, *RCT* randomized controlled trial, *VISA*-*A* Victorian Institute of Sports Assessment—Achilles, *VAS* visual analog scale, *US* ultrasound, *A*-*P* anterior–posterior, *LSI* Limb Symmetry Index, *NPRS* numerical pain rating scale, *mph* miles per hour

##### Reaching Pre-Injury Activity/Sports Level, with the Ability to Perform Training and Matches Without Limitations

The majority of studies used terminology such as ‘return to/resume previous activity/sports level’ [7, 8, 17, 19–22], ‘return to pre-injury activity/sports level’ [6, 23–26], or ‘return to the original activity/sports level’ [27–29] to define RTS. This finding was also reported in the included studies as ‘return to full (sports) activity’ [8, 21, 30–32], ‘return to full training schedule without limitations’ [28, 33], and ‘return to competition’ [16, 23].

##### Absence of Pain

When defining RTS, a few authors described ‘absence of pain’ as follows: ‘pain-free return to activity’ [34], ‘return to running without pain’ [35], or ‘return the patient to the desired level of activity without residual pain’ [36].

##### Recovery

In terms of recovery, terminology used to define RTS included ‘risk of re-injury’ (e.g. ‘safe return to sport while minimizing the risk of recurrent injury’ [37], ‘returning to activity and avoiding repeated injury’ [38], and ‘time to recovery’, which was described as ‘swift return’ [38] or ‘recovery time should be as short as possible’ [36].

#### Criteria

**Fig. 3:**
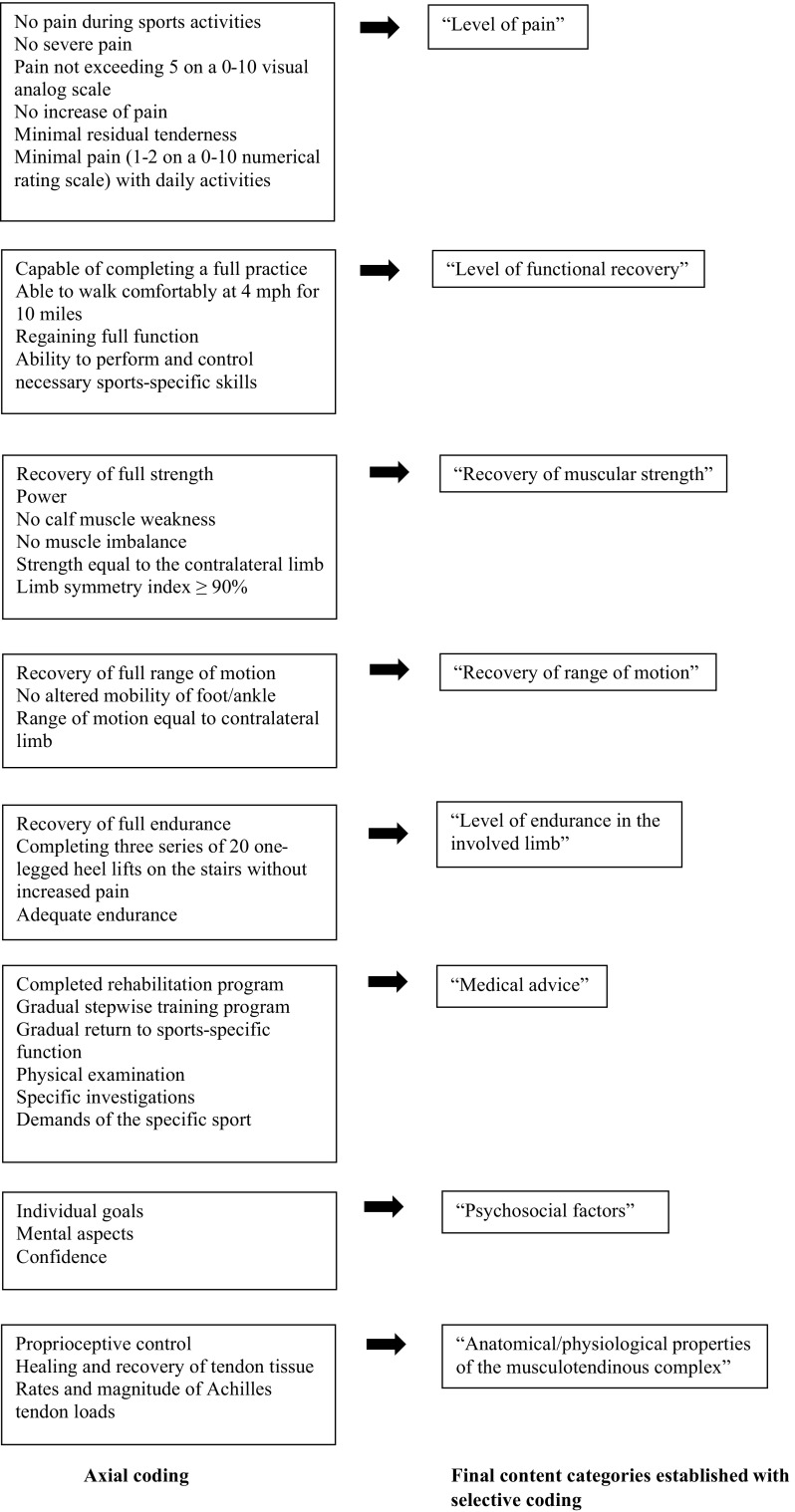
Axial coding and selective coding of the content analysis for criteria used for return to sport after midportion Achilles tendinopathy

Nineteen studies (54%) reported on one or more criteria for RTS after midportion AT (Table 1). Open coding resulted in different tentative labels, which were categorized during the axial coding phase. The final selective coding phase resulted in eight content categories (Fig. 3).

##### Level of Pain

Large variation was seen in the included studies with regard to pain as a criterion for RTS. Some studies reported a complete absence of pain as a criterion for RTS, whereas other studies accepted a certain level of pain. One study reported that pain during sports activities should not exceed 30 mm on a 0–100 mm visual analog scale (VAS) [22], while other studies stated that daily activities should be pain-free [35] or with minimal pain (1–2 on a 0–10 numerical pain rating scale) [8] before RTS can be considered.

##### Level of Functional Recovery

Within the included studies, multiple aspects of functional recovery were described as criteria for RTS after AT. Nicola and El Shami reported that return to running should not be considered until one is able to walk comfortably at 4.0 miles per hour (mph) for 10 miles per week [35], whereas Werd stated that “RTS decisions should be based on […] the ability of the athlete to perform the necessary skills of the sport without restriction” [38].

##### Recovery of Muscular Strength

In multiple studies, recovery of muscular strength was described as a criterion for RTS. Silbernagel and Crossley explicitly described that calf muscle weakness should be addressed before RTS [8], but other studies did not explicate the muscle groups that should be addressed.

One study reported a limb symmetry index of 90% or more as a guideline for RTS [39], while another study stated that recovery of strength to a level equal to the contralateral limb should be achieved [38]. No clear description was given of how muscle strength should be assessed.

##### Recovery of Range of Motion

In four studies, range of motion was reported as an RTS criterion for AT, with one study specifying this as ‘mobility of the foot and ankle complex’ [8]. Werd used the contralateral limb as reference value (‘equal to the contralateral limb’) [38], whereas other studies provided a more general description, such as ‘full range of motion’ [37].

##### Level of Endurance of the Involved Limb

Endurance was addressed as an RTS criterion for AT in four of the included studies. Wetke et al. stated that jumping and running activities should be ceased until an athlete can perform three sets of 20 one-legged heel lifts on the stairs (without increased pain) [40].

Neither the required level of endurance nor the preferred measurement method were clearly specified in the other studies [23, 37, 41].

##### Medical Advice

Several studies described that rehabilitation or a gradual stepwise training protocol should be completed prior to RTS [8, 23, 38], however, the exact measurement method was not clearly described. In the study by Biedert et al., physical examination and specific tests were also mentioned as RTS criteria for AT [42]; however, these were not further specified.

##### Psychosocial Factors

Psychosocial factors as criteria for RTS after AT were mentioned in one study [42]. The authors described that RTS depends on individual goals and mental aspects, but they did not further specify these factors.

##### Anatomical/Physiological Properties of the Musculotendinous Complex

In three of the included studies, anatomical/physiological properties of the musculotendinous complex, specified as ‘structural healing’ [42], ‘healing and recovery of the tendon tissue’ [8] and ‘proprioceptive control’ [41], were reported as criteria for RTS after AT. It was not clearly described how these properties were measured, e.g. whether imaging was used to determine the recovery of tendon tissue.

## Discussion

RTS is an important goal for many athletes suffering from midportion AT, and the decision to RTS may be influenced by many factors. This qualitative systematic review aimed to describe how successful RTS after midportion AT is defined, and which criteria are used to support the RTS decision. Of the 35 studies included in this review, 91% provided a definition, and only 54% reported criteria for RTS after AT. We found large variation in definitions and criteria for RTS within the different studies. Using a content analysis approach, we aimed to discover content categories that serve as umbrella terms for the definition of, and criteria for RTS after midportion AT.

### Definitions

Our content analysis approach identified three distinct content categories used to define successful RTS. Predominantly, we found that ‘pre-injury activity/sports level, with the ability to perform training and matches without limitations’ seemed to be an important term. We also found that ‘absence of pain’ and ‘recovery’ (minimal risk of re-injury or other injuries, and time to recovery) were other important terms used to define RTS after midportion AT.

In a recent consensus statement on RTS after sports injuries in general [4], it was stated that an RTS definition should, at a minimum, describe the type of sport and the sports level that is pursued. Many studies referred to the pre-injury level of sport of the involved athletes, but, unfortunately, this level of sport was often not clearly described. Lack of clear description impedes comparison of pre-injury to post-injury RTS rates; therefore, it will be beneficial to encourage studies to explicitly define the pre-injury sport and level of participation of their athletes. Ideally, this should be rated at baseline, or at least early during the intervention, to minimize recall bias of the participants.

Our results further show that, besides the type and level of sport, other relevant terms are also used to define RTS in the current AT literature. These terms were related to symptom level, time to recovery, and risk of re-injury. This implies that merely returning to a certain level of sport is not enough; RTS should also be achieved in a timely manner and with minimal risk of re-injury.

### Criteria

In total, 54% of the included studies described criteria for the RTS decision, but large variation in these criteria was found. Using content analysis, we were able to define eight final content categories: (1) level of pain; (2) level of functional recovery; (3) recovery of muscle strength; (4) recovery of range of motion; (5) level of endurance of the involved limb; (6) medical advice; (7) psychosocial factors; and (8) anatomical/physiological properties of the musculotendinous complex.

Many studies described the level of pain as an important criterion for RTS. Seven studies stated that ‘no pain’ should be present before RTS after midportion AT [21, 31, 35, 37, 38, 43, 44], whereas others used less specific and subjective terms, such as minimal or mild pain/discomfort [19, 25, 31, 43] or no severe pain in the tendon [7, 19]. Silbernagel and Crossley specified that the level of pain during daily activities should not exceed 2 on a 0–10 numerical pain scale before an athlete is allowed to return to running or jumping activities [8]. Beyer et al. also quantified the maximum level of pain that was allowed before RTS after AT [22], but they specified it as pain during sports activities and the level was slightly higher than the level used by Silbernagel and Crossley (i.e. 30 mm on a 0–100 mm VAS).

There is no doubt that pain is an important symptom of AT; in particular, morning pain/stiffness is a hallmark of AT. Morning pain/stiffness is considered as a useful clinical indicator of recovery [16] and has been included as part of the Victorian Institute of Sports Assessment—Achilles (VISA-A) questionnaire, which is considered a valid and reliable tool to evaluate AT symptoms [45]. Remarkably, none of the included studies explicitly described (absence of) morning pain/stiffness as a criterion for RTS. Furthermore, none of the studies used questionnaires such as the VISA-A as a criterion for RTS. It may be useful to investigate the possible role of the VISA-A in the decision to RTS among athletes with midportion AT, and to determine a cut-off score (e.g. ≥ 90 points [46]) as a required criterion for this decision.

Although many other criteria to support RTS after AT were described in the 35 included studies, it was remarkable that most of these criteria lacked essential information; the relevant body part was not described, no information on the preferred measurement method was given, or clear quantification or cut-off points were lacking. Regarding strength, for instance, studies reported information such as ‘balance of strength and flexibility’ [41] or ‘when full strength has returned’ [37]. Only one study explicitly described the relevant muscle group (i.e. calf muscle) [8], and only the study by Silbernagel et al. reported a limb symmetry index of 90% [39], which is often used as a reference for RTS in clinical practice. Furthermore, the vast majority of studies lacked information on which muscle groups should be tested (e.g. calf muscles, or all muscle groups of the lower extremity), what strength tests should be performed (e.g. isometric or isokinetic), which deficit between the injured and uninjured limb is considered acceptable, and how this could be measured. This lack of information applied to most of the criteria found in this review. This obviously may result in a large variety of measures being used, thereby impeding the clinician’s ability to make a well-considered and evidence-based decision on RTS. Additionally, it hampers comparison of RTS rates between different interventions for AT. Thus, we strongly encourage that studies comprehensively describe their criteria for RTS, and define clear cut-off values if possible. Furthermore, it would be of great interest if studies also reported the time to RTS as this is of much importance for clinicians and other stakeholders involved in RTS decision-making.

### Comparison with Other Findings

To our knowledge, this is the first systematic review investigating definitions and criteria for RTS in athletes with midportion AT, which limits the comparison with other findings. In the consensus statement on RTS after sports injuries, published by Ardern et al. [4], RTS was described as a process using three elements: (1) return to participation; (2) return to sport; and (3) return to performance. We believe that this categorization of relevant elements has some advantages compared with our findings regarding the definition of RTS. In our review, we found ‘pre-injury level of activity/sports, with the ability to perform training and matches without limitations’ to be an important term for defining successful RTS, but this appears to refer to the end stage of a rehabilitation process. Using the proposed approach by Ardern et al. [4], RTS is viewed more as a continuum, suggesting that earlier in the process of rehabilitation, athletes may be active in their sport, albeit at a lower level and less intensity.

The consensus statement of Arden et al. further suggested that the rate of RTS after AT varies between 10 and 86% after 12 weeks of treatment [4]. The authors blame the variety of activity levels for the large variation in RTS rates. At present, we think that the lack of an unambiguous definition may also be responsible for this large variation; if studies interpret RTS differently, this poses difficulty in comparing the success rates for RTS.

Our review attempted to synthesize RTS after temporarily ceasing sports activities. This was in line with the findings of several studies, which reported that up to 72% of athletes with AT need to cease their sports activities due to ongoing symptoms [29, 32]; however, research has demonstrated that completely ceasing sports activities may not be necessary. This point of view was based on a randomized controlled trial comparing two groups suffering from midportion AT [47]. The first group was allowed to engage in sports activities during the first 6 weeks of rehabilitation, using a pain-monitoring model. They were instructed that pain during sports activities should not exceed 5 on a 0–10 VAS, and that pain and stiffness in the Achilles tendon was not allowed to increase from week to week. The comparison group did not participate in Achilles tendon-loading sport for 6 weeks. As clinical improvement between both groups did not significantly differ, the authors concluded that continuing sports activities during rehabilitation using a pain-monitoring model is justified [47]. Although continuing sports activities using a pain-monitoring model may have advantages over temporary interruption (e.g. retaining tendon loading capacity and a positive effect on general health and psychological well-being), this decision should be made on an individual basis and should consider factors such as level of symptoms and psychological factors [48].

In a recent review of RTS after a rupture of the Achilles tendon [49], the authors concluded that 80% (range 18.6–100%) of athletes returned to sport approximately 6 months after the injury. However, interestingly, both rate and time differed between the included studies that clearly described definitions and measures of return to play, and those studies that did not provide a description of how RTS was assessed [49]. These findings are in line with our results, namely that there was a large variation in how RTS is defined, and many studies did not provide sufficient information on the type of measures that should be used to support the RTS decision. Therefore, we strongly advise both clinicians and researchers to achieve consensus, not only on a uniform definition for RTS after AT but also to define what measures (physical tests, performance tests, questionnaires, psychological factors, imaging) should be included in order to make the RTS decision process more efficient and successful. As many criteria are inter-related, it would be worthwhile to consider grouping them together with respect to clinical purpose. In future research, this may be addressed by performing a Delphi consensus strategy, similar to what was recently done for RTS after hamstring injuries [50].

### Strengths and Limitations

A strength of this review is that it was conducted in accordance with the PRISMA guidelines, which enhances its methodological quality. Additionally, we made no restrictions on study design in our selection criteria. While this may also be regarded as a limitation of the study, we feel that this decision maximized the chance of finding relevant literature on RTS after AT.

Our study also has some limitations that need to be addressed. First, a considerable proportion of studies (*n* = 10) could not be obtained in full text, despite serious efforts to contact the corresponding author of these studies (e-mail, ResearchGate) to obtain a copy. These studies may have used different definitions and/or criteria for RTS, which could obviously have influenced our results. Second, although we did not place limitations on study design, we only included studies investigating the effects of physiotherapeutic interventions. Therefore, we do not know whether studies on medication, injection, or operative treatments used different definitions and/or criteria.

## Conclusions

This qualitative systematic review revealed a large variation within AT research in how RTS is defined and which criteria should be used to support the RTS decision. This limits the clinician’s ability to make a well-considered RTS decision, and also hampers the comparison of RTS rates in different intervention studies. Using a content analysis approach, this systematic review showed that RTS may be defined according to the pre-injury level of sports (including both training and matches), but also with terms related to the absence of pain and recovery.

Currently, RTS decisions for midportion AT seem to be based on multiple criteria, which are all related to level of pain, level of functional recovery, muscular strength, range of motion, endurance, medical advice, psychosocial factors, and anatomical/physiological properties of the Achilles tendon. It was remarkable that, for most of the criteria we identified, no clear operationalization was given, which limits their practical usability. Therefore, there is an urgent need for future research aiming to reach consensus on how RTS after midportion AT should be defined, and what criteria should be used to support the decision on RTS.

## Electronic supplementary material

Below is the link to the electronic supplementary material.

Supplementary material 1 (DOCX 17 kb)
