# Supplementary material for: Return to Sport in Athletes with Midportion Achilles Tendinopathy: A Qualitative Systematic Review Regarding Definitions and Criteria
Source: Sports Med. 2017 Dec 16;48(3):705–23. doi: 10.1007/s40279-017-0833-9 (PMC5808052; doi:10.1007/s40279-017-0833-9)
Supplement: Supplementary file 1 — Supplementary material 1 (DOCX 17 kb) [file 40279_2017_833_MOESM1_ESM.docx]

**Electronic Supplementary Material Appendix S1. Search strategies for databases.**

| Database | Search string |
| --- | --- |
| PubMed | *(((“Achilles tendon”[Mesh] OR Achilles[tiab] OR Achillodynia[tiab] OR “Calcaneal tendon”[tiab] OR “Tendo Achillis”[tiab])) AND (Tendinopathy[Mesh] OR Tendinopathy[tiab] OR Tendinopathies[tiab] OR Tendinosis[tiab] OR Tendinitis[tiab] OR “Tendon injuries”[Mesh] OR “Tendon injuries”[tiab] OR “Tendon injury”[tiab])) AND (Sports[Mesh] OR “Sport activity”[tiab] OR “Sport activities”[tiab] OR “Sports activity”[tiab] OR “Sports activities”[tiab] OR “Sporting activity”[tiab] OR “Sporting activities”[tiab] OR “Tendon loading activity”[tiab] OR “Tendon loading activities”[tiab] OR “Provoking activities”[tiab] OR Competition[tiab] OR Match[tiab] OR Training[tiab] OR Practice[tiab] OR Rehabilitation[Mesh] OR Rehabilitation[tiab] OR “Recovery of function”[Mesh] OR “Return to sport”[Mesh] OR Return[tiab] OR Returning[tiab] OR Resume[tiab] OR Resuming[tiab] OR Resumption[tiab] OR “Previous level”[tiab] OR Engage[tiab] OR Engaging[tiab] OR Restart[tiab] OR Restarting[tiab] OR Continue[tiab] OR Continuing[tiab] OR “Carry on”[tiab])* |
| EMBASE (MEDLINE) | *(‘Achilles tendinitis’/exp OR ‘Achilles tendinitis’:ti,ab OR ‘Achilles tendon’/exp OR Achilles:ti,ab OR Achillodynia:ti,ab OR ‘Calcaneal tendon’:ti,ab OR ‘Tendo Achillis’:ti,ab) AND (Tendinitis/exp OR Tendinopathy:ti,ab OR Tendinopathies:ti,ab OR Tendinosis:ti,ab OR ‘Tendon injury’/exp OR ‘Tendon injuries’:ti,ab) AND (‘Return to sport’/exp OR ‘Return to sport’:ti,ab OR Sport/exp OR ‘Sport activity’:ti,ab OR ‘Sport activities’:ti,ab OR ‘Sports activity’:ti,ab OR ‘Sports activities’:ti,ab OR ‘Sporting activity’:ti,ab OR ‘Sporting activities’:ti,ab OR ‘Tendon loading activity’:ti,ab OR ‘Tendon loading activities’:ti,ab OR ‘Provoking activities’:ti,ab OR Competition/exp OR Competition:ti,ab OR Match:ti,ab OR Training/exp OR Training:ti,ab OR Practice:ti,ab OR Rehabilitation/exp OR Rehabilitation:ti,ab OR Convalescence/exp OR ‘Recovery of function’:ti,ab OR Return:ti,ab OR Returning:ti,ab OR Resume:ti,ab OR Resumption:ti,ab OR ‘Previous level’:ti,ab OR Engage:ti,ab OR Engaging:ti,ab OR Restart:ti,ab OR Restarting:ti,ab OR Continue:ti,ab OR Continuing:ti,ab OR ‘Carry on’:ti,ab)* |
| Cochrane | *“Achilles tendinopathy”:ti,ab OR Achilles:ti,ab OR Achillodynia:ti,ab OR “Calcaneal tendon”:ti,ab OR “Tendo Achillis”:ti,ab AND Tendinopathy:ti,ab OR Tendinopathies:ti,ab OR Tendinosis:ti,ab OR Tendinitis:ti,ab OR “Tendon injury”:ti,ab OR “Tendon injuries”:ti,ab AND “Sport activity”:ti,ab OR “Sport activities”:ti,ab OR “Sports activity”:ti,ab OR “Sports activities”ti,ab OR “Sporting activity”:ti,ab OR “Sporting activities”:ti,ab OR “Tendon loading activity”:ti,ab OR “Tendon loading activities”:ti,ab OR “Provoking activities”:ti,ab OR Competition:ti,ab OR Match:ti,ab OR Training:ti,ab OR Practice:ti,ab OR Rehabilitation:ti,ab OR “Recovery of function”:ti,ab OR Return:ti,ab OR Returning:ti,ab OR Resume:ti,ab OR Resuming:ti,ab OR Resumption:ti,ab OR “Previous level”:ti,ab OR Engage:ti,ab OR Engaging:ti,ab OR Restart:ti,ab OR Restarting:ti,ab OR Continue:ti,ab OR Continuing:ti,ab OR “Carry on”:ti,ab* |
| CINAHL | *MH (“Achilles tendinopathy” OR “Achilles tendon”) OR TI (“Achilles tendinopathy” OR “Achilles tendon” OR Achillodynia OR “Calcaneal tendon” OR “Tendo Achillis”) OR AB (“Achilles tendinopathy” OR “Achilles tendon” OR Achillodynia OR “Calcaneal tendon” OR “Tendo Achillis”) AND MH (Tendinopathy OR “Tendon injuries”) OR TI (Tendinopathy OR Tendinopathies OR Tendinosis OR Tendinitis OR “Tendon injury” OR “Tendon injuries”) OR AB (Tendinopathy OR Tendinopathies OR Tendinosis OR Tendinitis OR “Tendon injury” OR “Tendon injuries”) AND MH (“Sports re-entry” OR Sport OR “Sporting events” OR Rehabilitation OR Recovery) OR TI (“Sport activity” OR “Sport activities” OR “Sports activity” OR “Sports activities” OR “Sporting activity” OR “Sporting activities” OR “Tendon loading activity” OR “Tendon loading activities” OR “Provoking activities” OR Competition OR Match OR Training OR Practice OR Rehabilitation OR Recovery OR Return OR Returning OR Resume OR Resuming OR Resumption OR Engage OR Engaging OR Restart OR Restarting OR Continue OR Continuing OR “Carry on”) OR AB (“Sport activity” OR “Sport activities” OR “Sports activity” OR “Sports activities” OR “Sporting activity” OR “Sporting activities” OR “Tendon loading activity” OR “Tendon loading activities” OR “Provoking activities” OR Competition OR Match OR Training OR Practice OR Rehabilitation OR Recovery OR Return OR Returning OR Resume OR Resuming OR Resumption OR Engage OR Engaging OR Restart OR Restarting OR Continue OR Continuing OR “Carry on”)* |
| PEDro | *Achilles tendinopathy AND sports* |
| Scopus | *TITLE-ABS-KEY (achilles OR achillodynia OR "Calcaneal tendon" OR "Tendo Achillis") AND TITLE-ABS-KEY (tendinopathy OR*  *tendinopathies OR tendinosis OR tendinitis OR "Tendon injury" OR "Tendon injuries") AND TITLE-ABS-KEY ("Sport activity" OR "Sport activities" OR*  *"Sports activity" OR "Sports activities" OR "Sporting activity" OR "Sporting activities" OR "Tendon loading activity" OR "Tendon loading activities" OR*  *"Provoking activities" OR competition OR match OR training OR practice*  *OR rehabilitation OR "Recovery of function" OR return OR returning*  *OR resume OR resuming OR resumption OR "Previous level" OR engage OR*  *engaging OR restart OR restarting OR continue OR continuing OR "Carry on")* |
